# Supplementary material for: Hydrodeoxygenation of Levulinic Acid to γ-Valerolactone over Mesoporous Silica-Supported Cu-Ni Composite Catalysts
Source: Molecules. 2022 Aug 24;27(17):5383. doi: 10.3390/molecules27175383 (PMC9458178; doi:10.3390/molecules27175383)
Supplement: Supplementary file 1 [file molecules-27-05383-s001.zip › molecules-1854078-supplementary.pdf]

Hydrodeoxygenation of levulinic acid to γ-valerolactone over mesoporous silica supported Cu-Ni composite catalysts

Margarita Popova[1*], Ivalina Trendafilova[2,3], Manuela Oykova[1], Yavor Mitrev[1], Pavletta Shestakova[1], Magdolna Mihályi[4], Ágnes Szegedi[4*]

[1] Institute of Organic Chemistry with Centre of Phytochemistry, Bulgarian Academy of Sciences, Acad. G. Bonchev Str., Bl. 9, 1113

[2] Laboratory of Inorganic Materials Chemistry, University of Namur, Namur, 5000, Belgium

[3] Namur Institute of Structured Matter, University of Namur, Namur, 5000, Belgium

[4] Research Centre for Natural Sciences, 1117 Budapest, Magyar tudósok körútja 2, Hungary

*Corresponding authors: Margarita.Popova@orgchm.bas.bg, szegedi.agnes@ttk.hu

Supplementary data

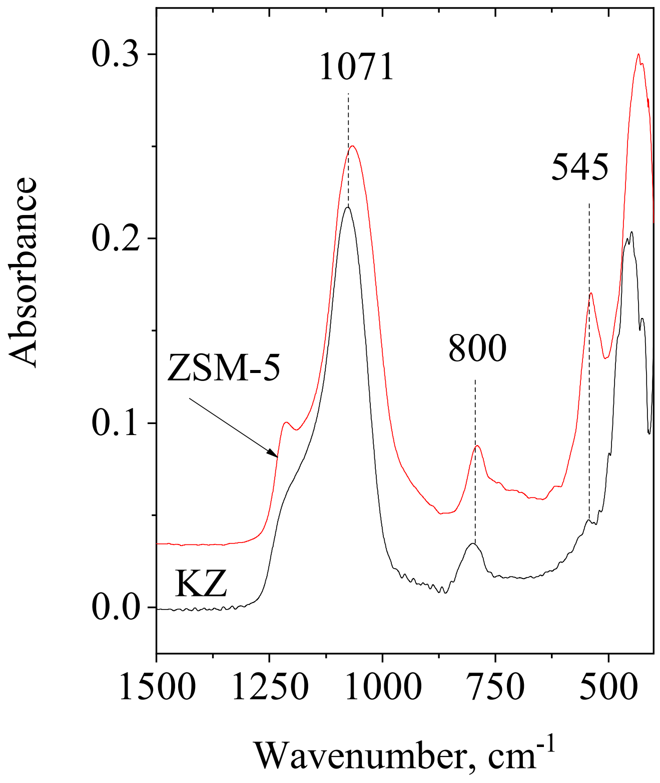

Figure S1 ATR FT-IR spectra of KZ mesoporous silica/zeolite composite compared to well crystallized ZSM-5 zeolite

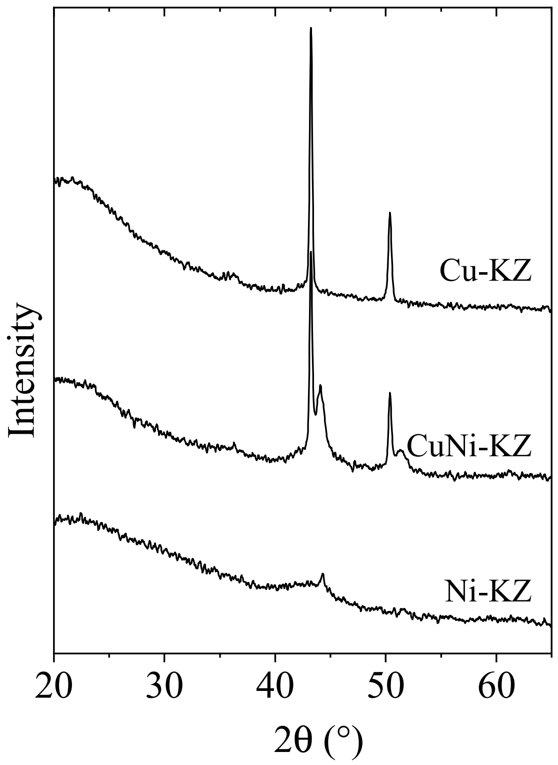

Figure S2. XRD patterns of the spent catalysts
